# Single crystal growth, optical absorption and luminescence properties under VUV-UV synchrotron excitation of type III Pr^3+^:KGd(PO_3_)_4_

**DOI:** 10.1038/s41598-020-63556-w

**Published:** 2020-04-21

**Authors:** Irina Adell, Maria Cinta Pujol, Rosa Maria Solé, Matthieu Lancry, Nadège Ollier, Magdalena Aguiló, Francesc Díaz

**Affiliations:** 1 Universitat Rovira i Virgili, Departament Química Física i Inorgànica, Física i Cristal·lografia de Materials i Nanomaterials (FiCMA‐FiCNA)‐EMaS, Campus Sescelades, E‐43007 Tarragona, Spain; 2 Institut de Chimie Moléculaire et des Matériaux d’Orsay, CNRS-Université Paris Sud, Université de Paris Saclay, Bât.410, 91405 Orsay, France; 3 Laboratoire des Solides Irradiés, CEA-CNRS-Ecole Polytechnique, Université Paris-Saclay, Palaiseau, France

## Abstract

Scintillator materials are widely used for a variety of applications such as high energy physics, astrophysics and medical imaging. Since the ideal scintillator does not exist, the search for scintillators with suitable properties for each application is of great interest. Here, Pr^3+^-doped KGd(PO_3_)_4_ bulk single crystals with monoclinic structure (space group: *P*2_1_) are grown from high temperature solutions and their structural, thermal and optical properties are studied as possible candidates for scintillation material. The change in the unit cell parameters as a function of the Pr^3+^ level of doping and temperature is studied. Differential thermal analysis reveals that KGd_0.942_Pr_0.058_(PO_3_)_4_ is stable until 1140 K. The 5*d*_3_, 5*d*_2_ and 5*d*_1_ levels of Pr^3+^ with respect to the ^3^H_4_ ground state are centred at 166, 196 and 218 nm, respectively, in this host. The luminescence of KGd_0.990_Pr_0.010_(PO_3_)_4_, by exciting these 5*d* levels, shows intense emissions centred at 256 and 265 nm from the 5*d*_1_ to ^3^F_3,4_ and ^1^G_4_ levels of Pr^3+^ with a short decay time of 6 ns. The ^6^P_3/2,5/2,7/2_ → ^8^S_7/2_ transitions of Gd^3+^ appear after exciting the 5*d* levels of Pr^3+^ and the 4 *f* levels of Gd^3+^, showing an energy transfer between Pr^3+^ and Gd^3+^.

## Introduction

Inorganic scintillation materials are widely used in a variety of applications in the field of particles and ionizing radiation detection such us medical imaging, dosimetry, nuclear physics and astrophysics^1^. Current research is focused on the search for new materials with improved scintillation properties^2^.

Ray imaging techniques for medical imaging include planar X-ray photography, computed tomography (CT) and positron emission tomography (PET). In the first of these, the number of UV-vis photons emitted by the scintillator material per energy unit of the incoming X-ray photons (light yield) should be high in order to decrease the X-ray dose to the patient. In CT, the stability of the light output should be as high as possible to achieve reliable images and therefore better diagnostics. In PET, a fast decay time of the UV-vis photons emitted by the scintillator is required for any improvement in spatial resolution and sensitivity, since this technique is based on a precise temporal measurement of two simultaneously emitted gamma photons at nearly 180° during a positron-electron annihilation process^3,4^.

Ce^3+^ and Pr^3+^ have been used as doping ions in the vast majority of the new single crystal scintillators reported over the last approximately 20 years because of the fast decay time of the 5*d* → 4 *f* radiative transitions (usually from 10 to 60 ns), together with the high quantum efficiency of these transitions at room temperature^2^. The scintillation properties of Ce^3+^- and Pr^3+^-doped garnets have been optimized by the growth of multicomponent doped hosts like (Gd,Lu)_3_Ga_3_Al_2_O_12_^5–8^. As regards aluminium perovskite crystals, fast lifetimes corresponding to the 5*d* → 4 *f* transitions of Ce^3+^ and Pr^3+^ ions doped in YAlO_3_ host have been obtained at around 18 and 8 ns, respectively^2^. Oxyorthosilicates have also been investigated because they have good scintillation properties, especially (Lu/Y)_2_SiO_5_ (LYSO) doped with Ce^3+^, which is used in PET imaging^2^. The Ce^3+^-doped LYSO compound was introduced in 2000 with the composition Ce:Lu_1.8_Y_0.2_SiO_5_ by Cooke *et al*.^9^. Lu_2_SiO_5_ (LSO) doped with Pr^3+^ has also been studied and the photoluminescence of the 5*d* → 4 *f* electronic transition at 273 nm shows a fast decay time of 6–7 ns. However, since the 5*d*_1_ level of Pr^3+^ in LSO is close to the conduction band, there is a degradation of the light yield of this scintillator at room temperature^10^.

In polyphosphate compounds, the photoluminescence of rare earth ions in LiLaP_4_O_12_ was studied by Blasse and Dirken^11^, with a Ce^3+^ concentration quenching being observed. The luminescence of LiY_0.9_Ce_0.1_P_4_O_12_ as a function of temperature was reported by Shalapska *et al*.^12^ with a decay time for the Ce^3+^ 5*d*_1_ → 4 *f* transition of 18.6 ns at room temperature. In the research carried out by Zhong *et al*. into Ce^3+^-doped MGdP_4_O_12_ (M = Li, Na, K, Cs)^13^, the energy transfer between Gd^3+^ and Ce^3+^ ions and its luminescence was studied and the energy level diagrams of the Gd^3+^ and Ce^3+^ in these compounds were put forward. Ce^3+^- and Pr^3+^-doped NaLa(PO_3_)_4_ were studied under VUV-UV excitation by Kang *et al*.^14^, showing decay times for the 5*d* → 4 *f* transitions of Ce^3+^ of 22.7–23.8 ns and of Pr^3+^ of 9.9–12.9 ns.

Type III KGd(PO_3_)_4_ is a monoclinic crystal with a non-centrosymmetric crystalline structure (space group: *P*2_1_) that has nonlinear optical properties similar to KH_2_PO_4_ (KDP)^15^ and a deep UV cut-off of its transparency window at 160 nm^16^. It is non-hygroscopic and chemically stable and its high hardness (close to quartz in the Mohs scale) means the surfaces can be polished to a good optical quality^17^. Considering the literature mentioned above, the 5*d* → 4 *f* transitions of Pr^3+^ in KGd(PO_3_)_4_ host is expected to have decay times faster than Ce^3+^ in the same host. Ce^3+^-doped KGd(PO_3_)_4_ single crystals have already been studied and show interesting luminescence characteristics for scintillator applications^18^. The aim of this paper is therefore to grow Pr:KGd(PO_3_)_4_ single crystals from high temperature solutions with different Pr^3+^ concentrations in order to characterize their thermal stability, study their optical absorption and luminescence properties under UV-VUV synchrotron excitation, and discuss their usefulness as a new scintillator material. However, it should first be mentioned that, a priori, the functionality of Pr:KGd(PO_3_)_4_ single crystals for some scintillator applications could be limited due to the 0.0117% natural abundance of the ^40^K radioisotope, which means an increase in the background counts is expected. Nevertheless, the ^40^K radioactive isotope together with others such as ^176^Lu, ^87^Rb, and ^138^La is present in some scintillator materials that are promising or already in use^19^.

## Results and discussion

### Bulk single crystal growth

Table 1 shows the crystal growth experiments carried out for different levels of Pr^3+^ doping and the crystals obtained.

**Table 1:** Crystal growth experiments for different Pr^3+^ doping levels and the crystals obtained.

[Pr_2_O_3_] / ([Gd_2_O_3_] + [Pr_2_O_3_]) in the solution [at. %]	Growth interval [K]	Crystal weight [g]	Crystal dimensions in a* × *b* × c* directions [mm]	Growth rate [× 10^−3^ g·h^−1^]
0.25	28.5	2.86	7.6 × 17.7 × 12.0	6.80
0.25	29.9	2.73	8.2 × 17.1 × 11.2	6.09
0.50	27.3	6.88	13.6 × 21.5 × 12.3	17.37
1.00	30	3.16	8.4 × 19.1 × 11.6	7.02
1.00	30	5.87	9.2 × 24.1 × 13.2	13.04
2.00	27.3	3.73	8.4 × 18.8 × 12.7	9.42
2.00	30	6.38	13.2 × 23.8 × 13.6	14.18
2.00	23.9	3.26	9.4 × 17.4 × 10.7	9.93
5.00	27.3	5.13	11.5 × 21.2 × 13.1	12.95
5.00	24.1	4.08	8.9 × 19.6 × 11.8	12.29
5.00	28.6	4.32	10.2 × 18.8 × 11.2	10.24

The saturation temperature of all solutions was around 993 K and no significant changes were observed with Pr^3+^ doping at the levels studied in this work. The saturation temperature is slightly higher than that previously reported for the crystal growth of Ce:KGd(PO_3_)_4_ crystals^18^ grown in similar experimental conditions. The crystals obtained were generally transparent, free of inclusions and cracks, and slightly greenish due to Pr^3+^ doping. The sizes obtained were a* × *b* × c* = 7.6–13.6 mm × 17.1–24.1 mm × 10.7–13.6 mm and the weights ranged from 2.7 to 6.9 g. As can be seen in Table 1, the crystal dimension in *b* direction was always higher than in a* and c* directions. This faster growth in the *b* crystallographic direction has already been reported by us for different doping KGd(PO_3_)_4_ crystals^16–18^ and is in agreement with the non-presence of the (010) crystalline face. The growth rate of the {hkl} form is inversely proportional to interplanar spacing *d*_hkl_ and the sequence in this crystal is *d*_001_ > *d*_100_ > *d*_020_. The chosen orientation of the seed also reinforces this behaviour, together with the thermal gradients in the solution. The crystal growth rate varies from 6.1 × 10^−3^ to 17.4 × 10^−3^ g·h^−1^.

**Figure 1:**
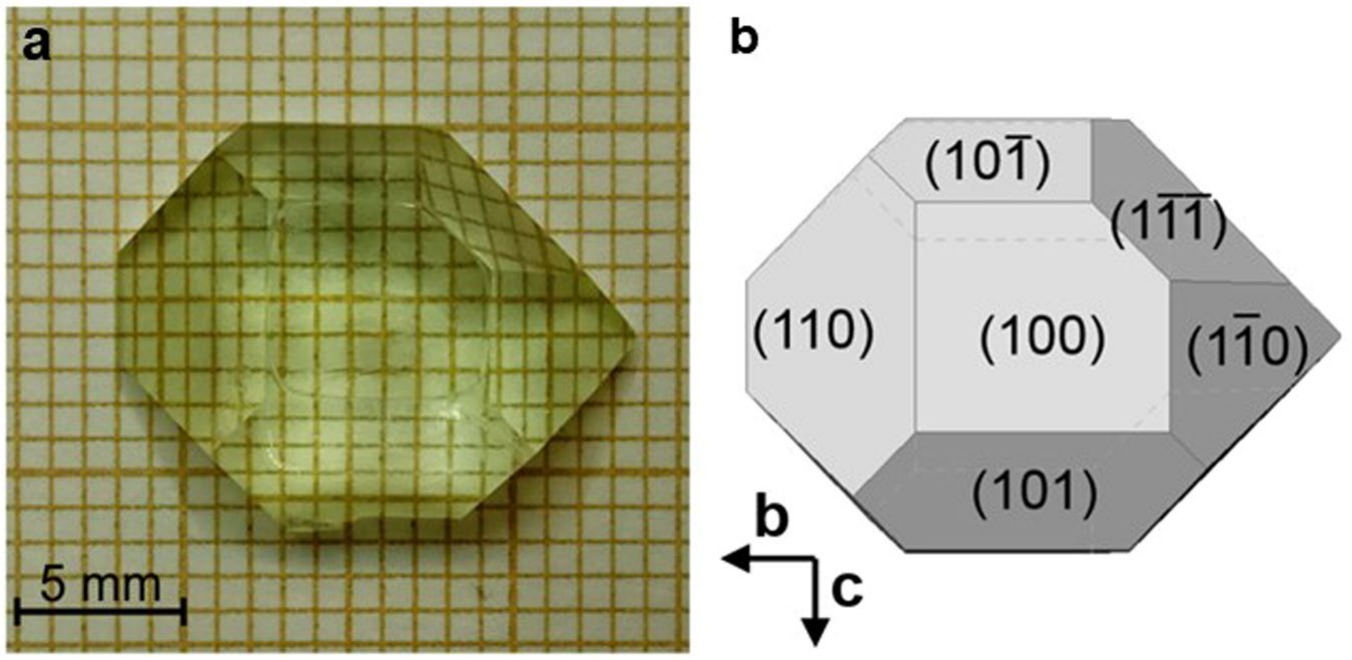
(**a**) As-grown single crystal of Pr:KGd(PO_3_)_4_, and (**b**) crystal scheme with the faces observed.

As an example, Fig. 1 shows an as-grown Pr:KGd(PO_3_)_4_ single crystal and a scheme of the crystal morphology in which the main faces of the crystal can be observed. These are generally {001}, {100}, {011}, {0–11}, {110}, {1–10}, {10-1}, {101} and {-1-11}.

Using the atomic percentage of each element present in the chemical compound as obtained from the EPMA (electron probe microanalysis) results, the chemical formula of each crystal was determined and the distribution coefficient of Pr^3+^ in KGd(PO_3_)_4_ calculated according to the formula *K*_Pr_ = ([Pr]/([Pr] + [Gd]))_crystal_/([Pr]/([Pr] + [Gd]))_solution_, where [Pr] and [Gd] are the Pr^3+^ and Gd^3+^ concentrations, respectively, in atomic % in the crystal and the solution. Table 2 shows, for the five doping levels studied, the Pr^3+^ atomic ratio with respect to Gd^3+^ in the crystal, the chemical formula, the number of Pr^3+^ ions per unit cell volume in the crystal and the distribution coefficient of Pr^3+^ (*K*_Pr_). For the crystals obtained from solutions with 2.00 and 5.00 atomic % of Pr^3+^ in the solution, the atomic percentage of each element was measured at several points along the a* and *c* crystallographic directions in a plate perpendicular to *b* crystallographic axis, obtaining up to about 85 results per sample. These results (see Fig. S1 in Supporting Information) showed that the variation of the measured values of Pr^3+^ atomic concentration along the crystallographic directions is of the same order as the error in the measurements. Therefore, the results indicate the uniformity of Pr^3+^ atomic concentration of the growing crystals along the a* and *c* crystallographic directions, up to 5.8 atomic % of Pr^3+^ in the crystal. In the samples obtained from solutions with 0.25, 0.50 and 1.00 atomic % of Pr^3+^ in the solution, the EPMA measurements were carried out far from the undoped KGd(PO_3_)_4_ seed, i.e. in the last stages of the crystal growth. Taking into account the values of the Pr^3+^ distribution coefficient in KGd(PO_3_)_4_ and their error, it can be observed that the Pr^3+^ distribution coefficient is not far from the unit in any of the concentrations studied. Besides, the results do not show any significant tendency for its value (*K*_Pr_) to decrease or increase as the level of Pr^3+^ doping in the solution increases.

**Table 2:** Compositional results for Pr:KGd(PO_3_)_4_. *K*_Pr_ denotes the distribution coefficient of the Pr^3+^ in the crystal.

[Pr]/([Gd] + [Pr]) atomic % ratio in the solution	[Pr] / ([Gd] + [Pr]) atomic ratio in the crystal	Chemical formula	Pr^3+^ concentration [cm^−3^]	*K*_Pr_
0.25	0.003 ± 0.001	KGd_0.997_Pr_0.003_(PO_3_)_4_	1.248 × 10^19^	1.2 ± 0.4
0.50	0.005 ± 0.002	KGd_0.995_Pr_0.005_(PO_3_)_4_	2.080 × 10^19^	1.0 ± 0.4
1.00	0.010 ± 0.002	KGd_0.990_Pr_0.010_(PO_3_)_4_	4.160 × 10^19^	1.0 ± 0.2
2.00	0.026 ± 0.001	KGd_0.974_Pr_0.026_(PO_3_)_4_	1.082 × 10^20^	1.30 ± 0.05
5.00	0.058 ± 0.001	KGd_0.942_Pr_0.058_(PO_3_)_4_	2.413 × 10^20^	1.16 ± 0.02

### Structural characterization

**Figure 2:**
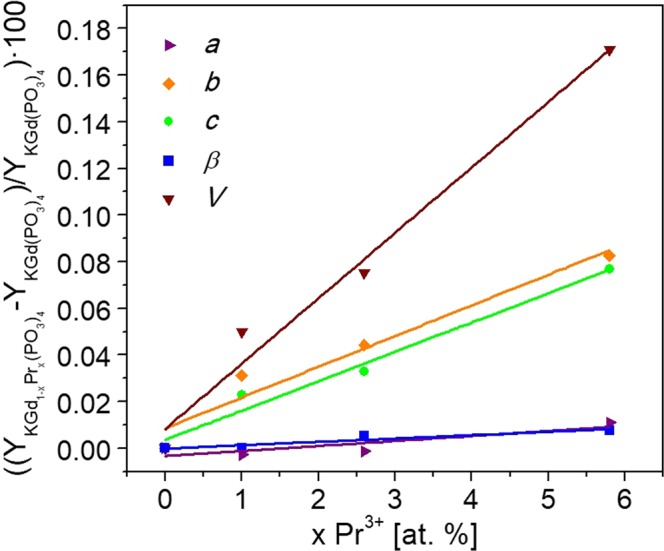
Evolution of the *a*, *b*, *c* and *β* unit cell parameters and unit cell volume of Pr:KGd(PO_3_)_4_ single crystals as a function of the praseodymium content in KGd(PO_3_)_4_.

X-ray powder diffraction analysis was carried out to study the evolution of the unit cell parameters of KGd_1-x_Pr_x_(PO_3_)_4_ depending on the Pr^3+^ doping concentration. The refinement of the unit cell parameters was carried out using the TOPAS program^20^. Table 3 shows the unit cell parameters of the crystals studied, while Fig. 2 shows the evolution of these parameters as a function of the praseodymium content in KGd(PO_3_)_4_. It can be seen that there is an ascending linear behaviour of the unit cell parameters on increasing the Pr^3+^ content in the crystals.

**Table 3:** The unit cell parameters and unit cell volume of Pr:KGd(PO_3_)_4_ single crystals at different Pr^3+^ doping concentrations.

Pr^3+^ at. % with respect to Gd^3+^ in KGd(PO_3_)_4_ crystal	*a* [Å]	*b* [Å]	*c* [Å]	*β* [°]	*V* [Å^3^]
0	7.2493 (3)	8.3466 (1)	7.9216 (1)	91.825 (2)	479.07 (2)
1.0	7.2491 (3)	8.3492 (1)	7.9234 (1)	91.825 (2)	479.31 (2)
2.6	7.2492 (3)	8.3503 (1)	7.9242 (1)	91.830 (2)	479.43 (2)
5.8	7.2501 (3)	8.3535 (1)	7.9277 (1)	91.832 (2)	479.89 (2)

As Table 3 and Fig. 2 show, the *a* and *β* parameters remain practically the same, the *b* and *c* parameters increase slightly and the unit cell volume clearly increases when the Pr^3+^ concentration in the crystal increases. This behaviour is expected because the ionic radius of Pr^3+^ with coordination VIII is higher than the ionic radius of Gd^3+^ with the same coordination (1.126 Å and 1.053 Å, respectively^21^).

### Thermal stability

**Figure 3:**
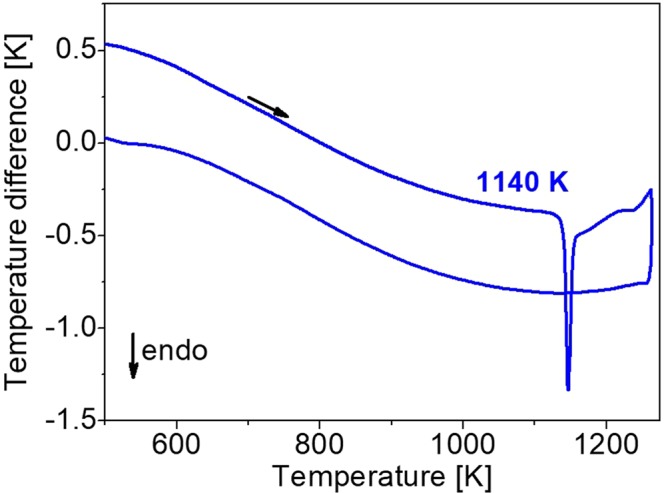
Thermogram of KGd_0.942_Pr_0.058_(PO_3_)_4_ in heating and cooling processes in the range 500–1273 K.

Figure 3 shows the thermogram obtained for KGd_0.942_Pr_0.058_(PO_3_)_4_ in both the heating and cooling processes in the range 500–1273 K. The weight change during the experiment was not significant.

An endothermic peak beginning at 1140 K can be observed in the heating process, which is attributed to the incongruent melting process of KGd_0.942_Pr_0.058_(PO_3_)_4_. This temperature is so similar to the 1142 K obtained for undoped KGd(PO_3_)_4_^22^ that it can be said that there is no appreciable difference in the incongruent melting temperature of KGd(PO_3_)_4_ with the Pr^3+^ doping, at least up to 5.8 atomic % of Pr^3+^ in the crystal. Meanwhile, no heat exchange in the cooling process of the sample was observed, which means that no crystalline phase transitions were produced during the process.

**Figure 4:**
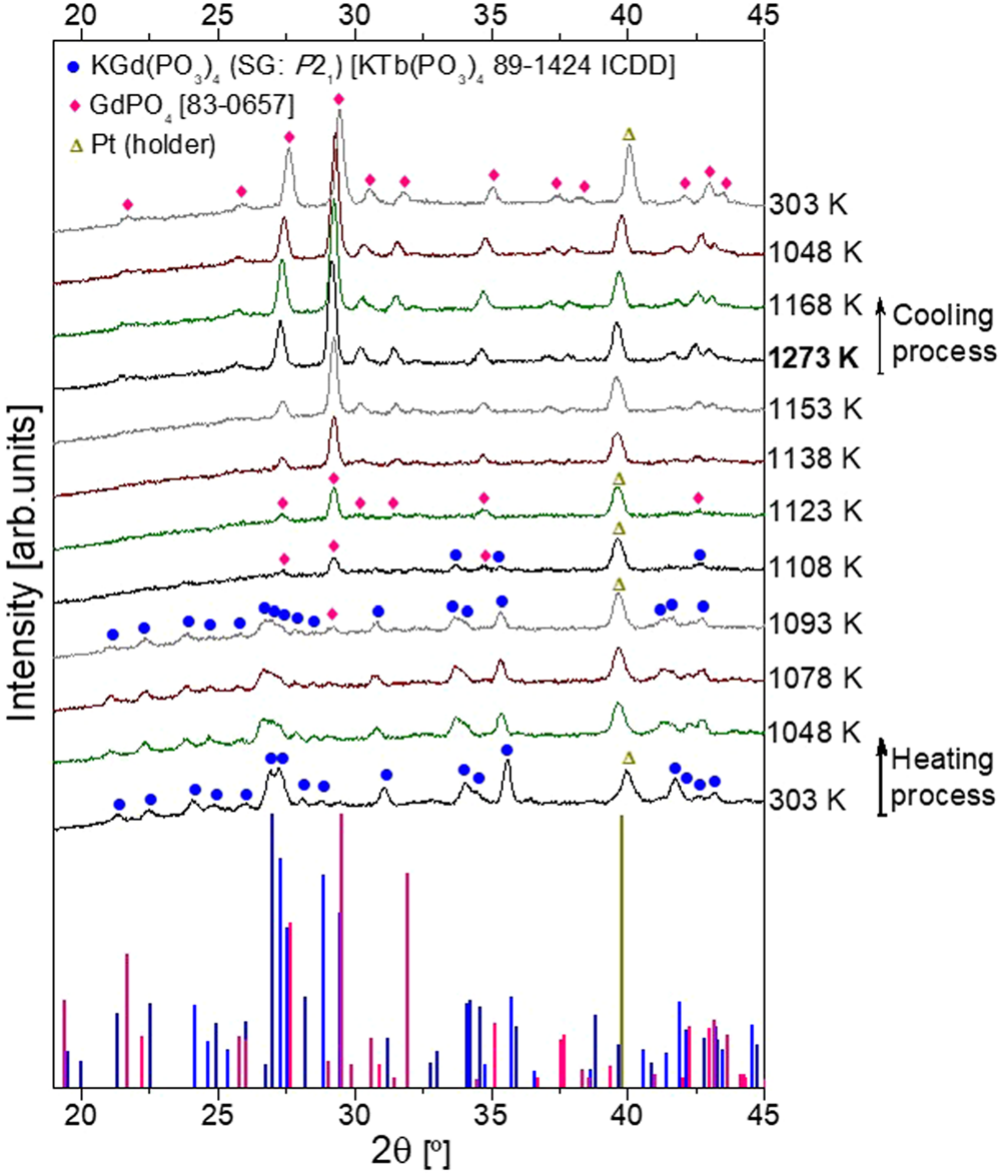
X-ray powder experimental diffractogram of KGd_0.942_Pr_0.058_(PO_3_)_4_ at room temperature, those selected at several temperatures describing its evolution with temperature in both the heating and cooling processes and three X-ray powder diffraction standard patterns. The labelling of the patterns is related to the temperature read in the central part of the sample support by the thermocouple.

Figure 4 shows the X-ray powder diffractogram of KGd_0.942_Pr_0.058_(PO_3_)_4_ at room temperature, its evolution with the temperature up to 1273 K and the cooling process up to room temperature. The temperatures written to the right of the graph are used as labels, since it is expected that the temperature distribution in the sample support during these measurements was not homogeneous. This could lead to a partial incongruent melting when the thermocouple of the diffractometer chamber indicated 1093 K.

The diffraction standard patterns of KTb(PO_3_)_4_ (89–1424 ICDD database^23^ and GdPO_4_ (83–0657 ICDD database^24^, together with a Pt diffraction peak (the sample holder was of Pt), are also shown in Fig. 4. The diffraction standard pattern of type III KTb(PO_3_)_4_ (space group: *P*2_1_) was used due to the non-existence of the type III KGd(PO_3_)_4_ powder diffraction standard pattern in the version of the ICDD database used. Hence at room temperature all diffraction peaks correspond to the monoclinic crystalline phase of type III KGd(PO_3_)_4_ together with a diffraction peak belonging to the Pt crystalline phase of the sample holder. Till 1078 K, there are no extra peaks of any other crystalline phase, and the diffraction peaks belonging to KGd(PO_3_)_4_ present a decrease in their sharpness and intensity, related to the loss of crystallinity. At 1093 K a small peak appeared at 29.2° and was identified as GdPO_4_. At 1108 K, the two crystalline phases (KGd(PO_3_)_4_ and GdPO_4_) are coexistent, and at 1123 K the KGd(PO_3_)_4_ crystalline phase has totally decomposed. In Ponceblanc *et al*.^25^, differences in the phase transition temperature of the same compound were also observed depending on both the heating rates and technique used. From 1093 to 1108 K, as the intensity of the type III KGd(PO_3_)_4_ peaks decreased, the intensity of the GdPO_4_ peaks increased. From 1123 K to 1273 K only the diffraction peaks of GdPO_4_ can be observed, meaning that this crystalline compound is stable at this range of temperatures. Throughout the cooling process from 1273 K until room temperature (see the last four diffractograms) there are no significant changes, so the GdPO_4_ remains stable. This means that the phase transition is not reversible, as expected for an incongruent melting, and in our case the solidification of the liquid phase leads to an amorphous phase.

Therefore, according to the differential thermal analysis and X-ray powder diffraction results, KGd_0.942_Pr_0.058_(PO_3_)_4_ decomposes at 1140 K into GdPO_4_ and liquid phase, which probably consisted of a mixture of phosphorus and potassium oxides, since the sample weight remained practically constant.

The studies on KGd(PO_3_)_4_^22^ and KYb_0.029_Gd_0.971_(PO_3_)_4_^17^ are comparable to that presented in our work. The results for the first compound show that KGd(PO_3_)_4_ decomposes irreversibly at 1142 K into Gd(PO_3_)_3_, GdPO_4_, Gd_2_P_4_O_13_ and an amorphous phase, and that at room temperature after the cooling process only GdPO_4_ remains. Regarding the second case, KYb_0.029_Gd_0.971_(PO_3_)_4_ decomposes irreversibly at 1130 K into Gd(PO_3_)_3_, at 1223 K Gd(PO_3_)_3_, GdPO_4_, Gd_2_P_4_O_13_, GdP_5_O_14_ and an amorphous phase coexist, and at room temperature after the cooling process the GdPO_4_ and Gd_2_P_4_O_13_ crystalline phases remain. Thus the difference in the thermal evolution observed for the KGd_0.942_Pr_0.058_(PO_3_)_4_ is that this crystal is decomposed into a unique crystalline compound, GdPO_4_, and a liquid phase. The intermediate Gd(PO_3_)_3_ crystalline compound observed in the previous studies is not observed in our case, and neither are the crystalline phases Gd_2_P_4_O_13_ and GdP_5_O_14_ from the previous works present in our case. Only the GdPO_4_ crystalline phase is observed to be stable till room temperature in all three studies^17,22^.

Another compound whose thermal decomposition has been studied in the literature is KLa(PO_3_)_4_^26^, which decomposes into La(PO_3_)_3_, LaPO_4_ and an amorphous phase containing phosphorus and potassium oxides. Thus, the thermal decomposition products of KLa(PO_3_)_4_ correlate with those of KGd(PO_3_)_4_ except for the fact that La_2_P_4_O_13_ is not present.

### Linear thermal expansion tensor

**Figure 5:**
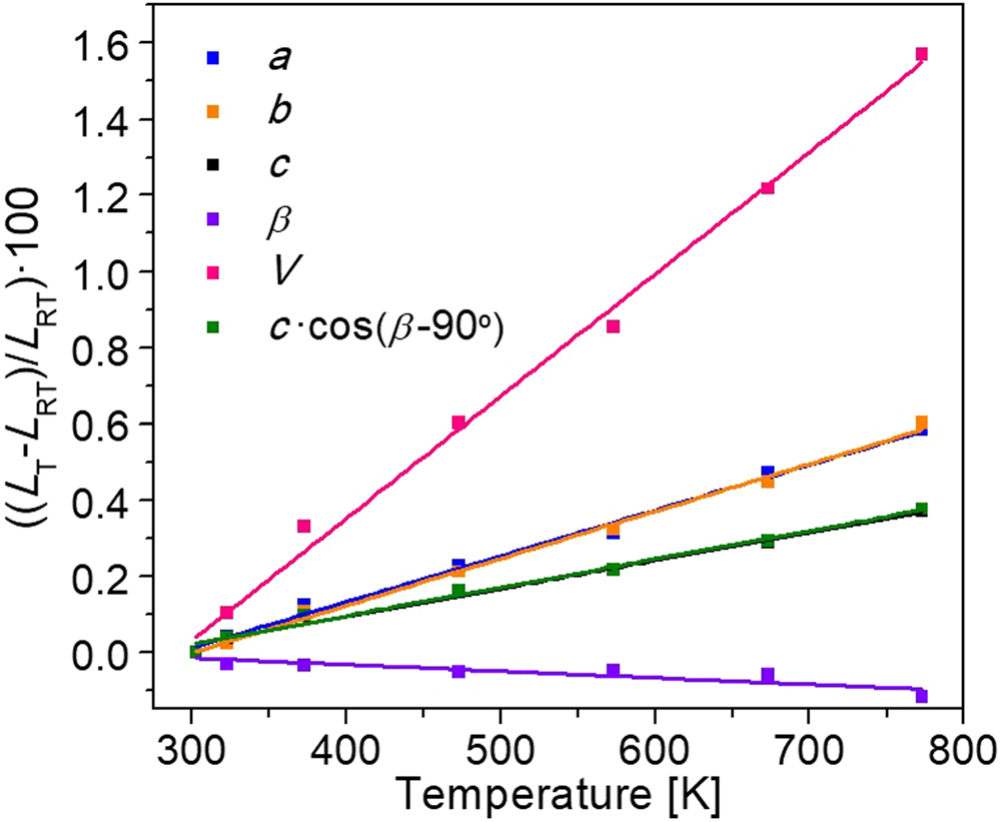
Evolution of the unit cell parameters of KGd_0.942_Pr_0.058_(PO_3_)_4_ from room temperature up to 773 K.

Bearing in mind the X-ray powder diffractograms measured in the range from room temperature up to 773 K and using the Le Bail method^27^, the unit cell parameters at different temperatures in the *P*2_1_ space group were refined. The parameters relating to goodness of fit are *R*_wp_ and *R*_exp_, whose values must fulfil the expression *R*_wp_ ≤ 2·*R*_exp_ for it to be considered that a good fit is obtained. In all cases these parameters are around *R*_wp_ = 21.06 and *R*_exp_ = 18.08. Table 4 shows the unit cell parameters of KGd_0.942_Pr_0.058_(PO_3_)_4_ at different temperatures, while Fig. 5 shows the relative thermal evolution of these parameters with respect to those at room temperature as a function of temperature. It can be seen that the unit cell parameters follow a linear trend. The *a* and *b* parameters clearly increase with temperature, as does the *c* parameter but in minor proportion, while the *β* parameter decreases slightly. From these results, the linear thermal expansion coefficients in each crystallographic direction can be calculated using the expression *α* = (Δ*L*/Δ*T*)/*L*_RT_, where Δ*L*/Δ*T* is the slope of the linear fit of the change of each unit cell parameter with the temperature, and *L*_RT_ is the unit cell parameter at room temperature, 303 K. The linear thermal expansion coefficients in the crystallophysical system *X*_1_∣∣*a*, *X*_2_∣∣*b* and *X*_3_∣∣c* are *α*_11_ = 12.00 × 10^-6^ K^−1^, *α*_22_ = 12.40 × 10^−6^ K^−1^, *α*_33_ = 7.46 × 10^−6^ K^−1^ and *α*_13_ = 1.45 × 10^−6^ K^−1^.

**Table 4:** Unit cell parameters and unit cell volume of KGd_0.942_Pr_0.058_ (PO_3_)_4_ at different temperatures.

T [K]	*a* [Å]	*b* [Å]	*c* [Å]	*β* [°]	*V* [Å^3^]
303	7.2486 (9)	8.3565 (8)	7.9306 (8)	91.871 (8)	480.12 (9)
323	7.2515 (9)	8.3584 (7)	7.9336 (6)	91.840 (7)	480.61 (8)
373	7.2576 (8)	8.3655 (7)	7.9382 (7)	91.838 (7)	481.71 (8)
473	7.2651 (8)	8.3742 (7)	7.9433 (7)	91.824 (7)	483.02 (8)
573	7.2712 (10)	8.3835 (9)	7.9476 (8)	91.827 (9)	484.22 (10)
673	7.2827 (11)	8.3939 (10)	7.9535 (10)	91.816 (10)	485.96 (11)
773	7.2909 (10)	8.4068 (9)	7.9600 (8)	91.764 (9)	487.66 (10)

**Figure 6:**
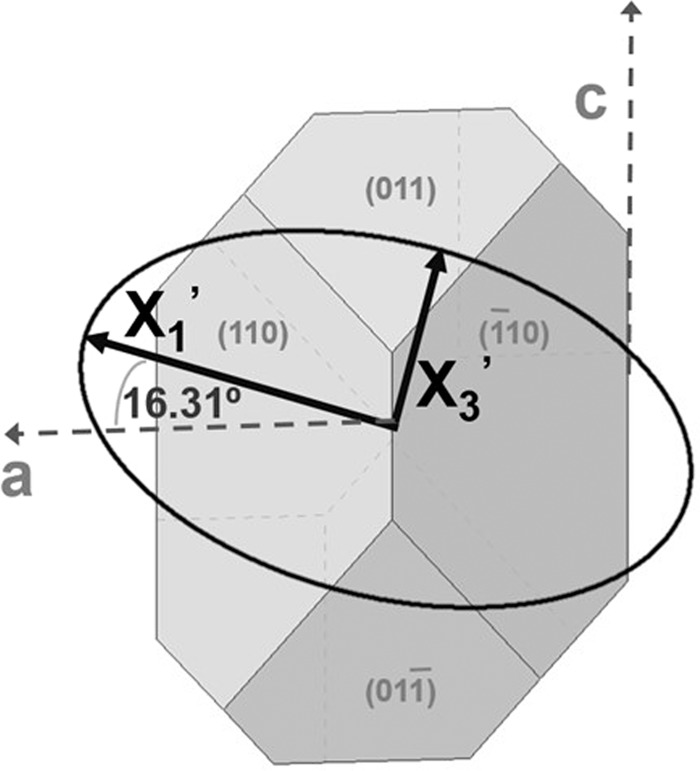
Linear thermal expansion ellipsoid of KGd_0.942_Pr_0.058_(PO_3_)_4_ at 303 K.

By diagonalizing the tensor, the thermal expansion values in the principal axes of the tensor *X*_1_’, *X*_2_’∣∣*b* and *X*_3_’ are obtained, these being *α*’_11_ = 12.43 × 10^−6^ K^−1^, *α*’_22_ = 12.40 × 10^−6^ K^−1^ and *α*’_33_ = 7.03 × 10^−6^ K^−1^. The *X*_1_’ axis is at 16.31° clockwise from the *a* axis, while *X*_3_’ is at 14.44° from the *c* axis with the positive *b* axis pointing toward the observer (see Fig. 6).

### Pr^3+^ spectroscopy in KGd(PO_3_)_4_ single crystals

**Figure 7:**
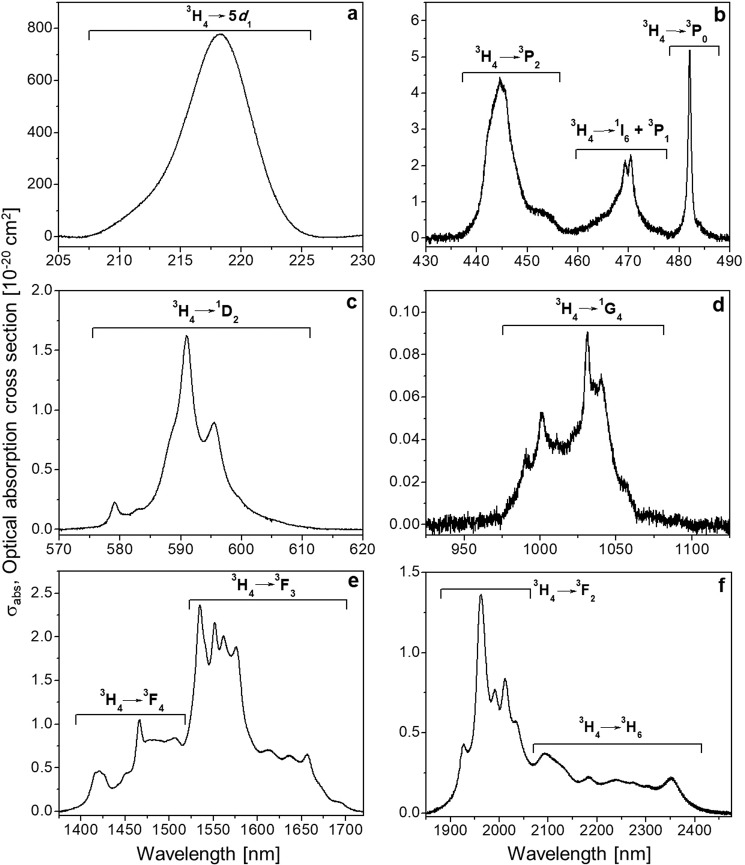
Unpolarized optical absorption cross sections of Pr:KGd(PO_3_)_4_ at room temperature. Light propagation direction is along the *b* axis in figure (**a**) and along the a* axis in figures (**b**–**f**). All labels indicate the electronic transitions of Pr^3+^.

*Optical absorption*. To identify the Pr^3+^ absorption bands and distinguish them from the Gd^3+^ bands in Pr:KGd(PO_3_)_4_, the optical absorption of an undoped sample of KGd(PO_3_)_4_ was measured (see Fig. S2 in Supporting Information). Figure 7 shows the unpolarized optical absorption cross sections of the ^3^H_4_ → 5*d*_1_ electronic transition and the 4 *f* → 4 *f* electronic transitions of Pr^3+^ ions in KGd(PO_3_)_4_ in the range 205 to 2475 nm at room temperature.

In Fig. 7a, a broad band centred at 218 nm (45872 cm^−1^, 5.69 eV) is observed, which corresponds to the electronic transition from the ^3^H_4_ ground state of Pr^3+^ to its lowest 5*d* level (5*d*_1_) in KGd(PO_3_)_4_. From the crystallographic point of view, there is only one site expected, with C_1_ point symmetry, for the Pr^3+^ and Gd^3+^ ions in the KGd(PO_3_)_4_ crystal^28^. Therefore all Pr^3+^ ions have the same crystal field, and consequently only one band for the ^3^H_4_ → 5*d*_1_ transition is expected. This transition of Pr^3+^ has been systematically studied in many hosts and it can be predicted by considering the study carried out by Dorenbos^29^. In this work, the value of the ^3^H_4_ → 5*d*_1_ transition of Pr^3+^ in phosphate hosts varies from 212 nm for LaP_3_O_9_^30^, through 222 nm for YPO_4_^30,31^, to 224 nm for YP_3_O_9_^30^. In addition, since the average energy difference of the first spin-allowed 4 *f* → 5*d* transition of Pr^3+^ (^3^H_4_ → 5*d*_1_) with respect to the transition of Ce^3+^ (^2^F_5/2_ → 5*d*_1_) in the same host^29^ is 12240 ± 750 cm^−1^ and because the ^2^F_5/2_ → 5*d*_1_ transition of Ce^3+^ in KGd(PO_3_)_4_ is centred at 302.5 nm (33058 cm^−1^, 4.10 eV)^18^, the expected position of the ^3^H_4_ → 5*d*_1_ transition of Pr^3+^ in KGd(PO_3_)_4_ is in the range 217.2–224.5 nm (46048–44548 cm^−1^, 5.71–5.52 eV). Therefore the experimental position of the ^3^H_4_ → 5*d*_1_ transition of Pr^3+^ in KGd(PO_3_)_4_ (218 nm) is consistent with the values found in other phosphates and within the calculated range.

The value of the optical absorption cross section of Pr^3+^ in KGd(PO_3_)_4_ for the ^3^H_4_ → 5*d*_1_ transition (at 218 nm) is about 780 × 10^−20^ cm^2^. This high value is expected since 4 *f* → 5*d* transitions are parity-allowed transitions.

It should be noted that, of the optical absorption measurements, only the 5*d*_1_ absorption band was identified out of all the 5*d*_x_ levels of Pr^3+^ in KGd(PO_3_)_4_. The reason for this is that, although the UV limit of the equipment used is 175 nm, the measurements were carried out in air atmosphere, and below 190 nm the air absorption hid the other 5*d*_x_ levels. The 5*d*_2_ and 5*d*_3_ energy levels, together with the 5*d*_1_ energy level already determined, of Pr^3+^ in KGd(PO_3_)_4_ were quantified in the UV-VUV synchrotron measurements (see next section) by studying the excitation spectra for several emission wavelengths.

Figure 7b–f show the optical absorption cross section of the Pr^3+^ 4 *f* → 4 *f* transitions in KGd(PO_3_)_4_ in the energy ranges 430–490, 570–620, 925–1125, 1375–1720 and 1850–2475 nm, in which the ^3^H_4_ → ^1^I_6_ + ^3^P_J_, ^3^H_4_ → ^1^D_2_, ^3^H_4_ → ^1^G_4_, ^3^H_4_ → ^3^F_4_ + ^3^F_3_ and ^3^H_4_ → ^3^F_2_ + ^3^H_6_ electronic transitions of Pr^3+^, respectively, were observed. Although the ^3^H_4_ → ^3^P_2_ pseudo-hypersensitive transition does not have the highest optical absorption cross section, its value of 4.4 × 10^−20^ cm^2^ is in the suitable range to be used for exciting 4 *f* → 4 *f* transitions for optical amplification applications.

### Optical emission

**Figure 8:**
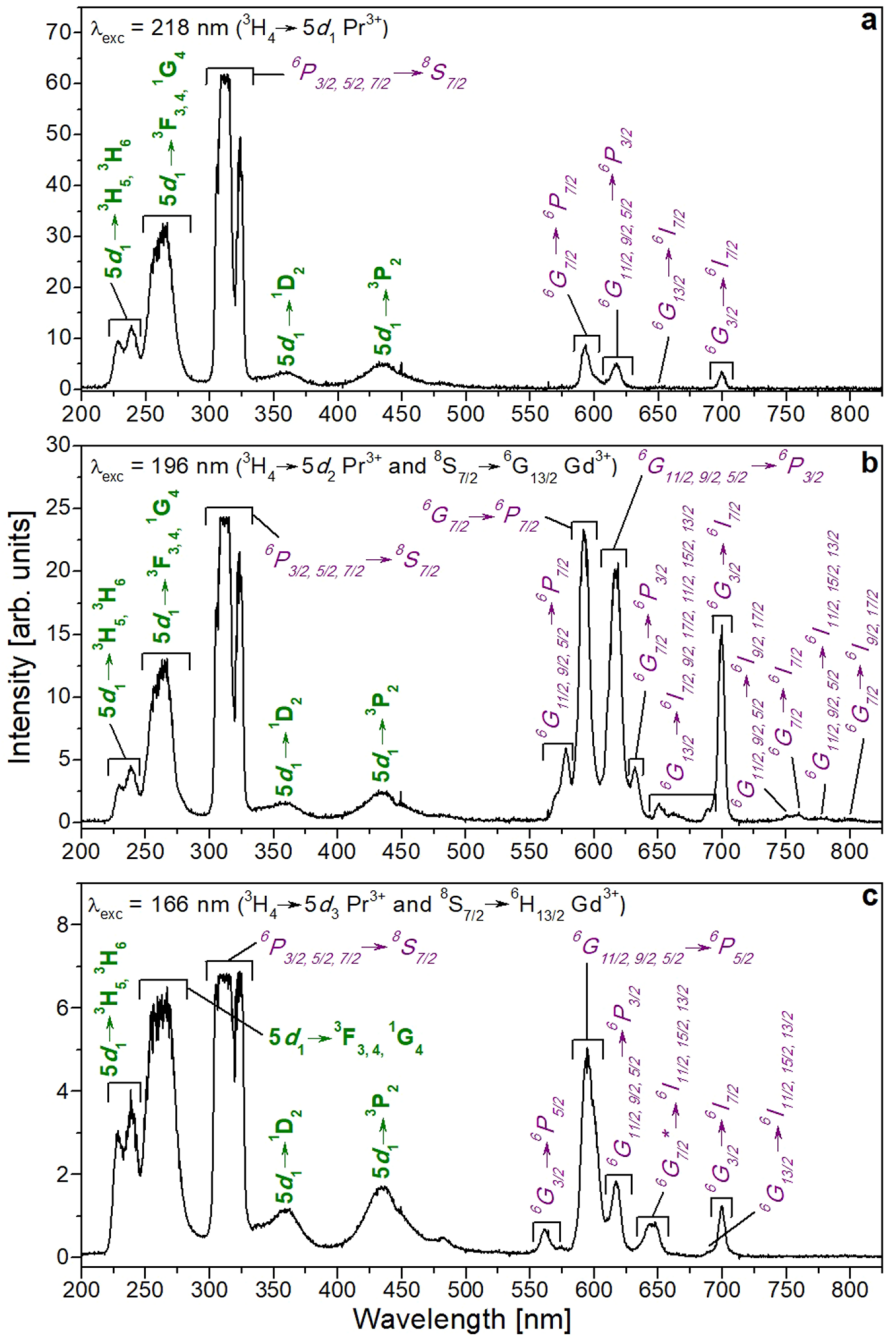
Optical emission spectra of KGd_0.990_Pr_0.010_(PO_3_)_4_ under (**a**) 218 nm, (**b**) 196 nm, and (**c**) 166 nm excitation. Labels in purple and italics indicate the electronic transitions of Gd^3+^ and labels in green and bold those of Pr^3+^.

Figure 8 shows the emission spectra of Pr:KGd(PO_3_)_4_ under λ_exc_ = 218 nm (45872 cm^−1^, 5.69 eV), 196 nm (51020 cm^−1^, 6.33 eV) and 166 nm (60241 cm^−1^, 7.47 eV) at room temperature.

In Fig. 8a, the most intense emissions centred at 305, 312 and 323 nm correspond to the ^6^P_3/2_ → ^8^S_7/2_, ^6^P_5/2_ → ^8^S_7/2_ and ^6^P_7/2_ → ^8^S_7/2_ transitions of Gd^3+^, respectively, obtained by exciting the 5*d*_1_ level of Pr^3+^ in KGd(PO_3_)_4_. This means that an energy transfer (ET) between Pr^3+^ and Gd^3+^ occurs. In other hosts such as (Gd,Lu)_3_Ga_3_Al_2_O_12_:Pr, energy transfers from 5*d* levels of Pr^3+^ to 4 *f* levels of Gd^3+^ were also observed^5^.

Also in Fig. 8a, parity-allowed transitions from the *d* to *f* levels of Pr^3+^, which are of great interest for scintillation applications, are also observed. These broad, intense bands correspond to the 5*d*_1_ → ^3^H_5_, 5*d*_1_ → ^3^H_6_, 5*d*_1_ → ^3^F_3,4_ and 5*d*_1_ → ^1^G_4_ electronic transitions of Pr^3+^, centred at 229, 239, 256 and 265 nm, respectively. The emission band corresponding to the electronic transition from the 5*d*_1_ level to the ^3^H_4_ ground state of Pr^3+^ does not appear, probably due to the self-absorption effect, as occurs in AREP_2_O_7_ hosts (A = Na, K, Rb, Cs; RE = Y, Lu)^32^. We should also note that the significantly weaker broad bands centred at 358 and 435 nm correspond to the 5*d*_1_ → ^1^D_2_ and 5*d*_1_ → ^3^P_2_ transitions. The most intense band originating in a 5*d* level is the broad band located around 256–265 nm, which corresponds to the overlapping of three electronic transitions, 5*d*_1_ → ^3^F_3,4_ and 5*d*_1_ → ^1^G_4_. By exciting the 5*d*_1_ level of Pr^3+^ at 210 nm in LiYF_4_ at 10 K, it can be observed how 5*d*_1_ → ^3^H_4_, 5*d*_1_ → ^3^H_5_, 5*d*_1_ → ^3^H_6_, 5*d*_1_ → ^3^F_3,4_ and 5*d*_1_ → ^1^G_4_ transitions appear at 220, 230, 245, 255 and 272 nm, respectively, while 5*d*_1_ → ^1^D_2_ and 5*d*_1_ → ^3^P_2_ transitions do not appear. It should also be noted that the band that corresponds to the 5*d*_1_ → ^3^H_5_ transition is the most intense, while that corresponding to the 5*d*_1_ → ^1^G_4_ transition is the least^33^. Under direct 4 *f* → 5*d*_1_ excitation (280 nm) of Pr^3+^ in Lu_3_Al_5_O_12_, Lu_3_Al_4_GaO_12_ and Lu_3_Al_3_Ga_2_O_12_ hosts, the emission bands corresponding to the 5*d*_1_ → ^3^H_J_ and 5*d*_1_ → ^3^F_J_ electronic transitions appear centred at 310 and 360 nm, respectively, with the first band being the most intense. The bands corresponding to the 5*d*_1_ → ^1^G_4_, 5*d*_1_ → ^1^D_2_ and 5*d*_1_ → ^3^P_J_ transitions do not appear. The emission peaks corresponding to some of the 4 *f* → 4 *f* electronic transitions of Pr^3+^ are insinuated in the visible range from 480 to 760 nm^34^. The emission spectrum of La_0.999_Pr_0.001_PO_4_ at 300 K under the direct 4 *f* → 5*d*_1_ excitation (193 nm) of Pr^3+^ shows intense emission bands centred at about 230, 240 and 255 nm corresponding to the 5*d*_1_ → ^3^H_4_, 5*d*_1_ → ^3^H_5_ and 5*d*_1_ → ^3^H_6_, ^3^F_2_ electronic transitions, respectively. Two very weak, broad emission bands appear centred around 375 and 440 nm corresponding to the 5*d*_1_ → ^1^D_2_ and 5*d*_1_ → ^1^I_6_,^3^P_J_ transitions. It should be noted that an emission peak centred at approximately 610 nm, with an intensity similar to the two previous bands, corresponds to the ^1^D_2_ → ^3^H_4_ electronic transition. The most intense emission bands correspond to the 5*d*_1_ → ^3^H_4_ and 5*d*_1_ → ^3^H_5_ transitions, and the least intense to the 5*d*_1_ → ^1^D_2_ transition^35^.

The weaker bands observed in the visible region of Fig. 8a are 4 *f* → 4 *f* transitions corresponding to Gd^3+^, which belong to the ^6^G_7/2_ → ^6^P_7/2_, ^6^G_11/2,9/2,5/2_ → ^6^P_3/2_, ^6^G_13/2_ → ^6^I_7/2_ and ^6^G_3/2_ → ^6^I_7/2_ transitions.

**Figure 9:**
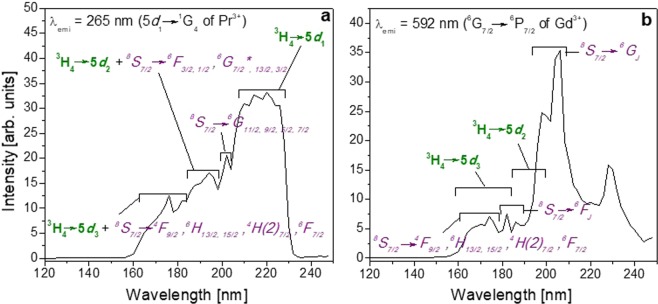
Excitation spectra of Pr:KGd(PO_3_)_4_ crystal under VUV radiation for (**a**) λ_emi_ = 265 nm and (**b**) λ_emi_ = 592 nm. Labels in purple and italics indicate the electronic transitions of Gd^3+^ and labels in green and bold those of Pr^3+^.

Information on the energy value of the 5*d*_2_ and 5*d*_3_ levels of Pr^3+^ in this host was obtained by studying the excitation spectra of Pr:KGd(PO_3_)_4_ for different emission wavelengths (see below in Fig. 9). In Fig. 8b, it can be seen that the emission spectrum of Pr:KGd(PO_3_)_4_ under λ_exc_ = 196 nm (5*d*_2_ level of Pr^3+^ and ^6^G_13/2_ level of Gd^3+^ excitation) is very similar to that obtained by exciting the 5*d*_1_ level as regards the intensity ratio of the emission bands in the range from 200 to 500 nm. The main difference obtained by exciting at 196 nm is in the 500–825 nm visible range, where the ^6^G_7/2_ → ^6^P_7/2_, ^6^G_11/2,9/2,5/2_ → ^6^P_3/2_, ^6^G_13/2_ → ^6^I_7/2_ and ^6^G_3/2_ → ^6^I_7/2_ transitions of Gd^3+^ are significantly intensified. Other new peaks appeared at 570–578, 632, 662, 689, 751, 759, 777 and 800 nm and correspond to the ^6^G_11/2,9/2,5/2_ → ^6^P_7/2_, ^6^G_7/2_ → ^6^P_3/2_, ^6^G_13/2_ → ^6^I_9/2,17/2_, ^6^G_13/2_ → ^6^I_11/2,15/2,13/2_, ^6^G_11/2,9/2,5/2_ → ^6^I_9/2,17/2_, ^6^G_7/2_ → ^6^I_7/2_, ^6^G_11/2,9/2,5/2_ → ^6^I_11/2,15/2,13/2_ and ^6^G_7/2_ → ^6^I_9/2,17/2_ transitions of Gd^3+^. Under excitation at 196 nm, the visible bands corresponding to the 4 *f* → 4 *f* electronic transitions of Gd^3+^ are more intense than the UV band corresponding to the 5*d* → 4 *f* electronic transitions of Pr^3+^. This could be related to a simultaneous energy transfer process from Pr^3+^ to Gd^3+^ and also direct Gd^3+^ excitation leading to a larger electronic population in the Gd^3+^ emitting levels. As in our work, photon cascade emissions of Gd^3+^ in the UV-Visible-near IR range were observed in GdBaB_9_O_16_ under ^8^S_7/2_ → ^6^G_J_ excitation (202 nm)^36^, and also under 195 nm excitation (^8^S_7/2_ → ^6^G_J_) in NaY_0.8_Gd_0.2_FPO_4_ and NaGdFPO_4_^37^.

Figure 8c shows the emission spectrum of Pr:KGd(PO_3_)_4_ under λ_exc_ = 166 nm (5*d*_3_ level of Pr^3+^ and ^6^H_13/2_ level of Gd^3+^ excitation). In this spectrum it is important to note that the main ^6^P_3/2,5/2,7/2_ → ^8^S_7/2_ electronic transitions of Gd^3+^ and the 5*d*_1_ → ^3^F_3,4_ and 5*d*_1_ → ^1^G_4_ emission bands of Pr^3+^ have a similar intensity. In the 500–825 nm range, the Gd^3+^ emissions located at 617 nm (^6^G_11/2,9/2,5/2_ → ^6^P_3/2_) and 700 nm (^6^G_3/2_ → ^6^I_7/2_) greatly decreased in intensity compared to the emission spectrum obtained by exciting at 196 nm and slightly decreased compared to the emission spectrum obtained by exciting at 218 nm. It should also be noted that three new emission peaks belonging to Gd^3+^ appear. These are centred at 561 nm (^6^G_3/2_ → ^6^P_5/2_), 595 nm (^6^G_11/2,9/2,5/2_ → ^6^P_5/2_) and 645 nm (^6^G_7/2_* → ^6^I_11/2,15/2,13/2_).

Finally, it is important to note that in Pr:KGd(PO_3_)_4_, no 4 *f* → 4 *f* transitions of Pr^3+^ were observed under excitations in the 120–248 nm range. The assignation of the Gd^3+^ and Pr^3+^ transitions was checked by comparing the Pr:KGd(PO_3_)_4_ emission spectra with those of the undoped KGd(PO_3_)_4_ excited at λ_exc_ = 218, 196 and 166 nm (see Fig. S3 in Supporting Information) and by consulting the Dieke’s diagram^38^, the extended Dieke’s diagram^39^, and the work carried out by Wegh *et al*.^40^ and by Yang *et al*.^36^.

In the excitation spectrum of Pr:KGd(PO_3_)_4_ crystal under VUV-UV radiation from 120 to 248 nm for λ_emi_ = 265 nm corresponding to the 5*d*_1_ → ^1^G_4_ electronic transition (see Fig. 9a), the excitation of the 5*d*_3,_ 5*d*_2_ and 5*d*_1_ levels of Pr^3+^ in KGd(PO_3_)_4_ is produced at wavelengths around 166, 196 and 218 nm, respectively. Some 4 *f* levels of Gd^3+^ were also excited giving rise to this Pr^3+^ emission, which could be explained by an energy transfer from Gd^3+^ to Pr^3+^.

As for the excitation spectrum for the Gd^3+^ emission at λ_emi_ = 592 nm (see Fig. 9b), although this emission could also be observed by excitation of the 5*d* energy levels of Pr^3+^ and the consequent energy transfer to Gd^3+^, it seems that it is more favoured when the ^6^G_J_ levels of Gd^3+^ are excited.

Given the calculations explained in our previous work on type III Ce:KGd(PO_3_)_4_ single crystals^18^, the energy of the exciton creation (*E*^ex^) and the approximate energy difference from the bottom of the conduction band to the top of the valence band (*E*_VC_) of the type III KGd(PO_3_)_4_ host were predicted at 7.57 eV (164 nm) and at 8.17 eV (152 nm), respectively. Therefore, as already mentioned in the previous work, the *E*^ex^ band could appear in the excitation spectra (Fig. 9), but it would not be appreciated due to an overlapping with the ^8^S_7/2_ → ^4^F_9/2_ transition of Gd^3+^.

**Figure 10:**
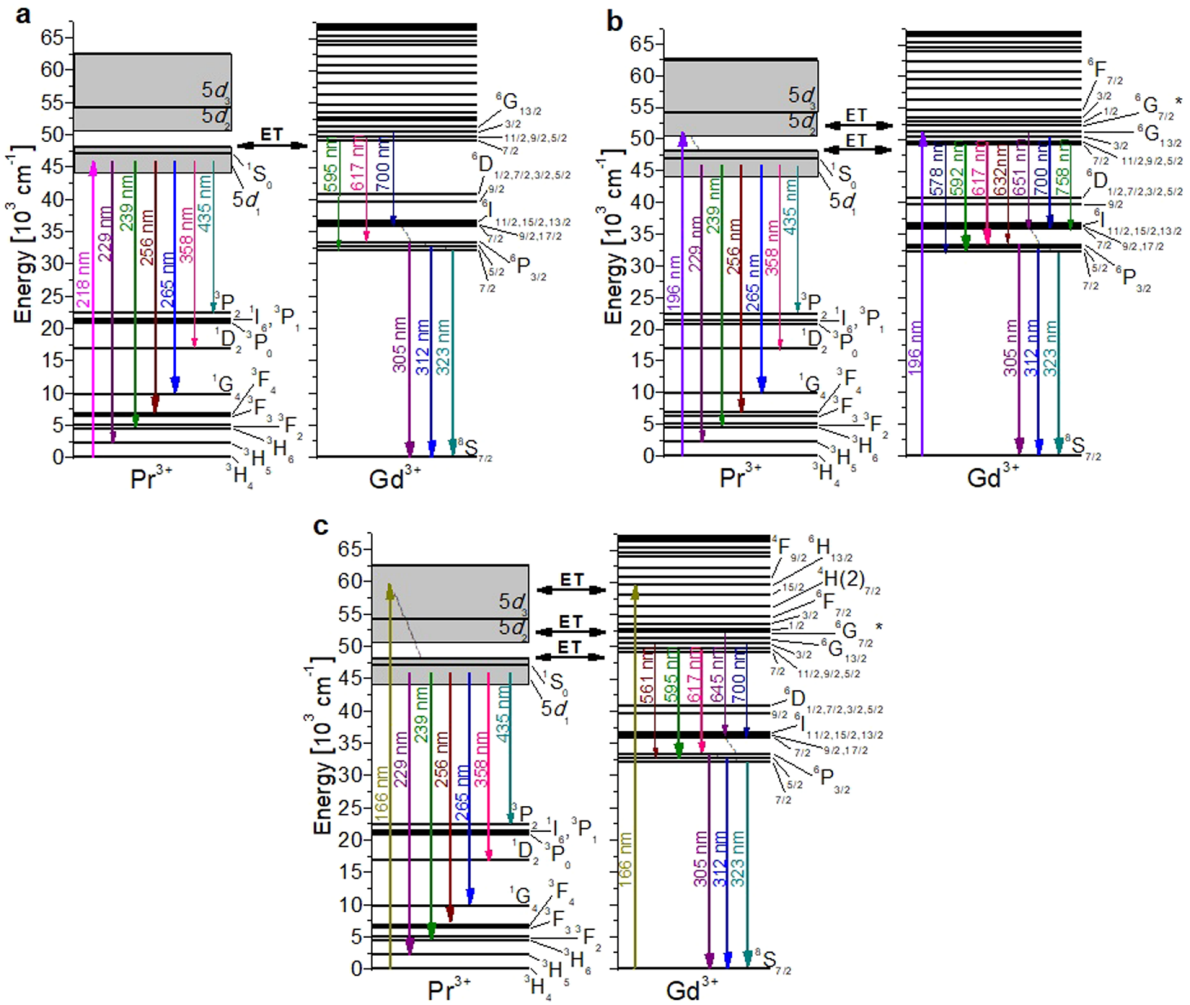
Energy level diagram of Pr^3+^ and Gd^3+^ in Pr:KGd(PO_3_)_4_ and emissions observed under excitation at (**a**) 218 nm, (**b**) 196 nm, and (**c**) 166 nm. The thickness of the arrows is related to the intensity of the emissions represented.

Figure 10 shows the energy levels scheme for Pr^3+^ and Gd^3+^ and the electronic transitions assigned to the observed emissions by excitation at 218, 196 and 166 nm in a KGd_0.990_Pr_0.010_(PO_3_)_4_ single crystal.

### Decay time measurements

**Figure 11:**
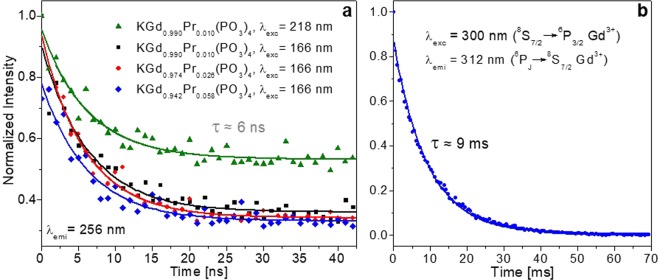
(**a**) Fast component of the decay curves of Pr:KGd(PO_3_)_4_ at different concentrations of praseodymium under excitations at 166 and 218 nm for λ_emi_ = 256 nm. (**b**) Slow component of the decay curve of KGd_0.942_Pr_0.058_(PO_3_)_4_ under excitation at 300 nm for λ_emi_ = 312 nm.

Time profiles were recorded in two time regimes to measure the fast and slow components of the decay curves of the emission at 256 nm and 312 nm, respectively. These curves are shown in Fig. 11. Pr:KGd(PO_3_)_4_ crystals were excited at 218 nm (^3^H_4_ → 5*d*_1_ of Pr^3+^) and 166 nm (^3^H_4_ → 5*d*_3_ of Pr^3+^ and ^8^S_7/2_ → ^6^H_13/2_ of Gd^3+^) under pulsed synchrotron radiation. In order to improve the photon flux reaching the sample, the excitation radiation was not exactly monochromatic but had a bandwidth of around 7%.

As can be seen in Fig. 11a, for the fast component all the decay curves can be fitted to single exponential decays with a time constant of around 6 ns. This value can be attributed to the lifetime of the 5*d*_1_ level of Pr^3+^ in KGd(PO_3_)_4_, which is significantly shorter than the lifetimes obtained in Ce:KGd(PO_3_)_4_^18^. The lifetimes obtained for the 5*d*_1_ emitting electronic state of Pr^3+^ in different hosts are usually longer than 6 ns, as observed in Table 5. The shortening of this lifetime in the KGd(PO_3_)_4_ host could be related to the energy transfer from Pr^3+^ to Gd^3+^, this being an additional depopulation channel through a non-radiative decay of the 5*d*_1_ emitting state of praseodymium.

**Table 5:** Values for the lifetime of the 5*d*_1_ level of Pr^3+^ in several hosts.

Compound	[Pr^3+^]	Lifetime [ns]	Ref.
LiPrP_4_O_12_	100 at. %	10.5	^44^
Pr:Lu_3_Al_5_O_12_	0.22–0.24 mol %	20.1	^45^
Pr:K_3_Lu (PO_4_)_2_	1 at. %	19.9–20.3	^46^
Pr(PO_3_)_3_	100 at. %	6	^47^
NaPr_0.998_Ce_0.002_P_4_O_12_	99.8 at. %	10.5	^48^
Pr:LiYF_4_	2%	16–19	^33^

It can also be seen that no significant changes in lifetime with the Pr^3+^ doping content of KGd(PO_3_)_4_ came about, at least up to 5.8 atomic % Pr:KGd(PO_3_)_4_. And no important quenching of the emission is expected due to the concentration effect, so no shortening of the lifetime. This can be seen in the long lifetime of the LiPrP_4_O_12_ phosphate (Table 5), where Pr^3+^ is not a doping element but a host element.

As previously stated, the emission at around 312 nm observed in Fig. 8 corresponds to some of the 4 *f* → 4 *f* electronic transitions of Gd^3+^. The decay time of these electronic transitions (lifetime could be in μs or ms) is significantly slower than those from the 5*d* energy levels and could not be measured at the DESIRS beamline (DESIRS-6.65 m Monochromator) due to the interpulse duration of 1118 ns of the synchrotron radiation. Figure 11b shows its slow decay time. By fitting to a single exponential decay, the lifetime obtained is 9 ms. As reported previously in the literature, the origin of this slow component could be attributed to the Gd^3+^ emission corresponding to the electronic transition ^6^P_J_ → ^8^S_7/2_ along with some contribution from the trapping effect, since the lifetime of the emitting ^6^P_7/2_ level of Gd^3+^ is of the same order (4.9 ms in NaY_0.80_Gd_0.20_PO_4_ and 6.36 ms in NaGd(PO_3_)_4_)^41,42^ as the 9 ms.

## Conclusions

Type III Pr^3+^-doped KGd(PO_3_)_4_ single crystals of up to 5.8 atomic % of Pr^3+^ substituting Gd^3+^ with high crystalline quality have been grown by the top seeded solution growth-slow cooling technique from self-flux solutions. It has been demonstrated that KGd_0.942_Pr_0.058_(PO_3_)_4_ is thermally stable up to 1140 K, where it suffers an irreversible decomposition into a unique crystalline compound, GdPO_4_, and a liquid phase. The *X*_1_’ principal axis of the thermal tensor of this crystal is at 16.31° clockwise from the *a* crystallographic direction when the positive *b* axis (parallel to the *X*_2_’ principal axis) is pointing toward the observer and the *X*_3_’ principal axis is at 14.44° clockwise from the *c* axis. The absorption bands corresponding to the ^3^H_4_ → 5*d*_1_, ^3^H_4_ → 5*d*_2_ and ^3^H_4_ → 5*d*_3_ electronic transitions of Pr^3+^ in KGd(PO_3_)_4_ are centred at 218, 196 and 166 nm, respectively. The ^1^S_0_ energy level of Pr^3+^ overlaps with the 5*d*_1_ level of the same ion in this host, preventing the non-radiative relaxation from the 5*d*_1_ level to the ^1^S_0_ energy level and the radiative relaxation between 4 *f* levels that would deteriorate the scintillation efficiency of these crystals. Under ultraviolet excitation, an intense, broad emission band located around 256–265 nm was observed in all grown crystals, corresponding to the 5*d*_1_ → ^3^F_3,4_ and 5*d*_1_ → ^1^G_4_ electronic transitions of Pr^3+^. The lifetime of the 5*d*_1_ level of the Pr^3+^ in type III KGd(PO_3_)_4_ was measured for the emission band centred at 256 nm and a lifetime of around 6 ns was obtained, which is of great interest for scintillator applications. In most cases this lifetime is shorter than the lifetime obtained for the same level of Pr^3+^ in other hosts. Moreover, the emission spectra show a broad band in the visible range corresponding to the 5*d*_1_ → ^3^P_2_ transition of Pr^3+^ with enough intensity under 218 nm excitation, which could be an appropriate transition for use in scintillator applications. It should also be noted that the ^6^P_3/2,5/2,7/2_ → ^8^S_7/2_ electronic transitions of Gd^3+^ were observed centred at 305, 312 and 323 nm by exciting the 5*d* levels of Pr^3+^, although it would be interesting to study whether the same behaviour occurs under X-ray excitation.

## Experimental

### Single crystal growth

Type III praseodymium-doped KGd(PO_3_)_4_ single crystals, with doping levels ranging up to 5 atomic % of Pr^3+^ substituting Gd^3+^ in the solution, were grown from self-flux solutions using the top seeded solution growth-slow cooling (TSSG-SC) technique. The growth solutions, with a weight of around 130 g, were placed in a platinum cylindrical crucible 50 mm in diameter and 50 mm in height. The initial reagents used were K_2_CO_3_ (99%), Gd_2_O_3_ (99.9%), Pr_2_O_3_ (99.9%) and NH_4_H_2_PO_4_ (≥99.0%). The compositions of the solutions, chosen according to the KGd(PO_3_)_4_ primary crystallization region in the K_2_O – Gd_2_O_3_ – P_2_O_5_ ternary system^22^, were K_2_O: ((1−x) Gd_2_O_3_ + x Pr_2_O_3_): P_2_O_5_ = 36: 4: 60, mol %, with x ranging from 0 to 0.05.

A platinum stirrer with a diameter of 18 mm, located at 12–14 mm below the solution surface and rotating at 55 rpm with a change of rotation direction every 50 s, was used to mix the solution. This stirring was needed because of the high level of dynamic viscosity of the growth solution, around 19 Pa·s^43^. An a* oriented KGd(PO_3_)_4_ seed was placed in contact with the surface of the solution at 12 mm from the solution centre, rotating with the same angular velocity as the stirrer. The use of this crystallographic direction in the KGd(PO_3_)_4_ seeds leads to the growth of high crystalline quality crystals^16,22^. The crystallographic *c* direction of the seed was oriented in radial direction, while its *b* direction was tangential to the rotation movement in order to achieve a good aerodynamic orientation of the crystal during its movement. Note that the morphology of this crystal usually presents an edge perpendicular to the *b* crystallographic direction, while it has natural {001} faces. With the aim of further improving the mixing of the solution, high thermal gradients (around 11 K·cm^−1^ in depth, with the hottest point at the bottom) were applied.

Once the solution was homogeneous, its saturation temperature was determined by accurately measuring the growth/dissolution rate of the KGd(PO_3_)_4_ seed depending on the temperature, which will then be used to start the growth of the single crystal. Beginning at the saturation temperature of the solution, cooling rates of 0.1 K·h^−1^ for the first 15 K and 0.05 K·h^−1^ for the next 10–15 K were applied to create supersaturation and grow the single crystal. At the beginning of the crystal growth experiments, the cooling rate was higher in order to initiate growth and not lose contact between the crystal seed and the solution. During this initial cooling rate regime, the supersaturation of the solution increases gradually because of the difficulty in mixing the solution due to its high viscosity. After decreasing the temperature of the furnace 15 K, a second cooling rate was applied that was slower than the first to avoid an additional increase in the supersaturation of the solution, since this could induce nucleation in different points of the solution and also inclusions of solution inside the crystals. The growth rate can be maintained even with a slower cooling rate due to the accumulated supersaturation of the solution and the larger crystal surface.

After finishing the thermal cooling ramps, the crystal was removed from the solution and maintained at a few mm above the surface of the solution while the furnace was cooled to room temperature at a rate of 20–25 K·h^−1^.

Electron probe microanalysis (EPMA) with wavelength dispersive spectrometry (WDS) was used to determine the Pr content of the crystals. In this non-destructive technique, an electron beam is focused on the sample and the characteristic X-rays emitted (specific to each element of the sample) are dispersed by crystals (WDS) before being recorded and compared with the emission of standard compounds containing the elements to be analysed. The X-rays of the sample and the standards are obtained under the same measurement conditions. The equipment used was a JEOL JXA-8230. The standards used were an undoped KGd(PO_3_)_4_ single crystal for K, Gd, P and O measurements and an REE-1 for determining the Pr concentration. An accelerating voltage of 20 kV and a current of 20 nA were applied, with measuring time of 10 s for K, P, Gd and O and 100 s for Pr peaks and 5 s and 50 s for background measuring, respectively. Kα lines of K, P and O and Lα lines of Gd and Pr were used. The dispersive crystals were PETJ for K, PETH for P, LDE1 for O, and LIFL for Gd and Pr measurements. The detection limit of Pr^3+^ was around 105 ppm.

### Structural characterization and thermal stability

The evolution of the unit cell parameters of Pr:KGd(PO_3_)_4_ with the Pr^3+^ content was studied by X-ray powder diffraction measurements, using a D5000 Siemens X-ray powder diffractometer in vertical θ-θ configuration with the Bragg-Brentano geometry. The X-ray diffraction patterns of undoped KGd(PO_3_)_4_ and 1, 2 and 5 atomic % Pr:KGd(PO_3_)_4_ in solution were obtained using Cu Kα radiation and recorded in the 2θ range from 10 to 70°. The measurements were made with a step size of 0.03° and a step time of 7 s. The unit cell parameters were refined using the TOPAS program^20^, the Le Bail method^27^ and the crystal data for undoped type III KGd(PO_3_)_4_ studied by Parreu *et al*.^28^ (171710 ICSD database).

The thermal stability of the KGd(PO_3_)_4_ doped with praseodymium was studied by X-ray powder diffraction. The equipment used was a Bruker-AXS D8-Discover diffractometer equipped with a Cu source, a parallel incident beam (Göbel mirror), a HI-STAR GADDS (general area detector diffraction system) and a MRI BTS-Solid temperature chamber with a platinum ribbon heating stage. The powder samples were placed in the centre (occupying an area of ~1 × 1 mm^2^) on the platinum ribbon. This stage was covered with a beryllium dome to maintain temperature. The sample was heated and cooled at a rate of 10 K·min^−1^. Diffraction patterns in the heating and cooling cycles were recorded every 15 K between 1048 and 1273 K and twice at room temperature, one diffraction pattern before the heating process and the other after the cooling process. The measurements were made in the 2θ range from 18 to 52° (one frame) with a detector-sample distance of 15 cm, an exposition time of 300 s per frame and a delay time of 60 s before each frame.

To complement the study of the thermal stability of the KGd(PO_3_)_4_ doped with praseodymium, differential thermal and thermogravimetric analyses (DTA-TGA) were performed using a TA Instruments SDT 2960 Simultaneous DSC-TGA. Al_2_O_3_ was used as the reference material, and the heating and cooling rates were at 10 K·min^−1^ with an air flux of 90 cm^3^·min^−1^.

The evolution of the unit cell parameters of the crystals grown from a 5 atomic % Pr:KGd(PO_3_)_4_ in solution with temperature was also studied by X-ray powder diffraction. The equipment was the same D5000 Siemens X-ray powder diffractometer previously used to study the Pr:KGd(PO_3_)_4_ unit cell parameters, but with an Anton-Paar HTK10 temperature chamber with a platinum ribbon heating stage. The sample was placed in the centre (occupying an area of ~9 × 5 mm^2^) on the platinum ribbon. The diffraction patterns were recorded at temperatures of 298, 323, 373, 473, 573, 673 and 773 K (in which the monoclinic *P*2_1_ crystalline phase of KGd(PO_3_)_4_ is stable), in the 2θ range from 10 to 70° with a step size of 0.03°, a step time of 5 s and a delay time of 300 s before each measurement. As before, the unit cell parameters were refined using the TOPAS program^20^, the Le Bail method^27^ and the crystal data for undoped type III KGd(PO_3_)_4_ studied by Parreu *et al*.^28^ (171710 ICSD database).

### Optical characterization

The bulk single crystals obtained were cut in plates perpendicular to the crystallographic a*, *b* and c* directions with a diamond saw. The plates were initially lapped and then polished with Al_2_O_3_ particle solutions to a size of 0.1 μm using a Logitech polishing machine. These plates were used for the optical absorption and emission studies. The unpolarized optical absorption of Pr^3+^ in KGd(PO_3_)_4_ was studied using a CARY 5000 UV-Vis-NIR spectrophotometer at room temperature in the wavelength range from 205 to 2475 nm, while the unpolarized optical absorption of undoped KGd(PO_3_)_4_ was studied in the wavelength range from 190 to 315 nm.

The emission spectroscopy was studied under vacuum ultraviolet-ultraviolet (VUV-UV) excitation in the wavelength range from 120 to 248 nm (10–5 eV). Experiments were performed in the DESIRS beamline at SOLEIL Synchrotron, France (proposal number 20151215, standard). The samples were placed in a vacuum chamber which can be evacuated to a pressure below 2 × 10^−5^ bars. A lithium fluoride window at the entrance of the vacuum chamber separates it from the synchrotron line. The monochromatized synchrotron light reached the sample at an angle of 90°. The emitted light from the sample was collected at 45°, focused with a silica lens and analysed with an Ocean Optics Jaz spectrometer (minimum spectral resolution 0.3 nm). The emission spectra were recorded in the range from 192 to 886 nm. To obtain the excitation spectra, the intensity obtained for a particular emission wavelength was plotted in front of the excitation wavelength in the excitation wavelength range from 120 to 248 nm.

Lifetime measurements were also carried out in the DESIRS beamline of SOLEIL Synchrotron, France (proposal number 20161324, standard) in a single bunch mode operation for pulsed radiation. The same configuration in the vacuum chamber as in previous measurements was used. The light was guided with an optical fibre to an ANDOR spectrograph (Shamrock 193i) with a grating of 150 lines·mm^−1^, coupled to an iStar Intensified Charge Coupled Device (ICCD) (model DH734-18F-03). When the level of vacuum in the chamber was lower than 2 × 10^−5^ bars, the window between this chamber and the synchrotron was removed in order to increase the photon flux reaching the sample.

The decay time of the 4 *f* → 4 *f* electronic transitions, which was significantly slower than those of the 5*d* energy levels, was measured with a Cary Eclipse fluorescence spectrophotometer.

## Supplementary information

Supplementary information.

## References

[1] Rodnyi, P. A. Scintillators requirements in various applications in Physical Processes in Inorganic Scintillators 41–51 (CRC Press, 1997).

[2] Nikl M, Yoshikawa A (2015). Recent R&D trends in inorganic single-crystal scintillator materials for radiation detection. Adv. Opt. Mater..

[3] Zyck, A. K. Luminescence properties of Ce^3+^, Pr^3+^ and Nd^3+^ activated scintillators for positron emission tomography (PET). Ph.D. Thesis. Utrecht University, Utrecht, Netherlands (2011).

[4] Waterstram-Rich, K. M. & Gilmore, D. PET instrumentation in Nuclear Medicine and PET/CT: Technology and Techniques 326–355 (Elsevier, 2017).

[5] Wu Y, Ren G (2013). Energy transfer and radiative recombination processes in (Gd,Lu)_3_Ga_3_Al_2_O_12_:Pr^3+^ scintillators. Opt. Mater..

[6] Tyagi M (2013). Effect of codoping on scintillation and optical properties of a Ce-doped Gd_3_Ga_3_Al_2_O_12_ scintillator. J. Phys. D: Appl. Phys.

[7] Kamada K (2015). Alkali earth co-doping effects on luminescence and scintillation properties of Ce doped Gd_3_Al_2_Ga_3_O_12_ scintillator. Opt. Mater..

[8] Wu Y, Ren G (2013). Effects of Gd/Lu ratio on the luminescent properties of Pr^3+^ activated (Gd,Lu)_3_Ga_3_Al_2_O_12_. ECS J. Solid State Sci. Technol..

[9] Cooke DW (2000). Crystal growth and optical characterization of cerium-doped Lu_1.8_Y_0.2_SiO_5_. J. Appl. Phys..

[10] Nikl, M., Vedda, A. & Laguta, V. V. Single-crystal scintillation materials in Springer Handbook of Crystal Growth (eds. Dhanaraj, G., Byrappa, K., Prasad, V. & Dudley, M.) 1663–1700 (Springer, 2010).

[11] Blasse G, Dirksen GJ (1982). The luminescence of broad-band emitters in LiLaP_4_O_12_. Phys. Status Solidi B.

[12] Shalapska T (2010). Crystal structure and luminescence properties of LiYP_4_O_12_:Ce^3+^ phosphor. J. Phys. Condens. Matter.

[13] Zhong J (2007). Effects of crystal structure on the luminescence properties and energy transfer between Gd^3+^ and Ce^3+^ ions in MGd(PO_3_)_4_:Ce^3+^ (M  =  Li, Na, K, Cs). J. Mater. Chem..

[14] Kang Y (2013). VUV-UV luminescence of Ce^3+^, Pr^3+^ doped and Ce^3+^-Pr^3+^ codoped NaLa(PO_3_)_4_. J. Lumin..

[15] Parreu, I. Crystal growth and characterization of ytterbium or neodymium doped type III-KGd(PO3)4. A new bifunctional nonlinear and laser crystal. Ph.D. Thesis. Universitat Rovira i Virgili, Tarragona, Spain (2006).

[16] Solé RM (2015). Growth, anisotropic spectroscopy and laser operation of the monoclinic Nd:KGd(PO_3_)_4_ crystal. J. Phys. D Appl. Phys..

[17] Parreu I (2007). Crystal growth and characterization of type III ytterbium-doped KGd(PO_3_)_4_: a new nonlinear laser host. Chem. Mater..

[18] Adell I (2018). Single crystal growth, optical absorption and luminescence properties under VUV-UV synchrotron excitation of type III Ce^3+^:KGd(PO_3_)_4_, a promising scintillator material. Sci. Rep..

[19] Nikl M (2006). Scintillation detectors for x-rays. Meas. Sci. Technol..

[20] XRD software –diffract suit TOPAS V4.2. Bruker, 2007.

[21] Shannon RD (1976). Revised effective ionic radii and systematic studies of interatomic distances in halides and chalcogenides. Acta Cryst.

[22] Parreu I (2005). Crystal growth, structural characterization, and linear thermal evolution of KGd(PO_3_)_4_. Chem. Mater..

[23] Xing Y, Hu NH, Zhou QL, Hong GY, Yue SY (1987). Wuli Huaxue Xuebao.

[24] Ni Y-X, Hughes JM, Mariano AN (1995). Crystal chemistry of the monazite and xenotime structures. Am. Mineral..

[25] Ponceblanc H, Millet JMM, Thomas G, Herrmann JM, Védrine JC (1992). Comparative study of polymorphic phase transition by differential thermal analysis, high temperature X-ray diffraction, and temperature programmed electrical conductivity measurements. Case study of mixed iron and cobalt molybdate. J. Phys. Chem.

[26] Shan P (2016). Crystal growth and optical characteristics of beryllium-free polyphosphate, KLa(PO_3_)_4_, a possible deep-ultraviolet nonlinear optical crystal. Sci. Rep..

[27] Le Bail A (2005). Whole powder pattern decomposition methods and applications: a retrospection. Powder Diffr..

[28] Parreu I, Carvajal JJ, Solans X, Díaz F, Aguiló M (2006). Crystal structure and optical characterization of pure and Nd-substituted type III KGd(PO_3_)_4_. Chem. Mater..

[29] Dorenbos P (2000). The 4f^*n*^ ↔ 4f^*n-1*^5d transitions of the trivalent lanthanides in halogenides and chalcogenides. J. Lumin..

[30] Mayolet, A. Etude des processus d’absorption et de transfert d’energie au sein de materiaux inorganiques luminescents dans le domaine UV et VUV. Ph.D. Thesis. Université de Paris XI, Paris, France (1995).

[31] Naik RC, Karanjikar NP, Narasimham NA (1981). X-ray excited optical luminescence spectrum of Pr-doped YPO_4_. Solid State Commun..

[32] Yuan JL (2007). VUV spectroscopic properties of Ce^3+^ and Pr^3+^-doped AREP_2_O_7_-type alkali rare earth diphosphates (A  =  Na, K, Rb, Cs; RE  =  Y, Lu). J. Lumin..

[33] van Pieterson L, Wegh RT, Meijerink A, Reid MF (2001). Emission spectra and trends for 4*f*^n-1^5*d* ↔ 4*f*^n^ transitions of lanthanide ions: Experiment and theory. J. Chem. Phys..

[34] Katelnikovas A, Bettentrup H, Dutczak D, Kareiva A, Jüstel T (2011). On the correlation between the composition of Pr^3+^ doped garnet type materials and their photoluminescence properties. J. Lumin..

[35] Srivastava AM (2011). The influence of the Pr^3+^ 4f^1^5d^1^ configuration on the ^1^S_0_ emission efficiency and lifetime in LaPO_4_. Opt. Mater..

[36] Yang Z, Lin JH, Su MZ, Tao Y, Wang W (2000). Photon cascade luminescence of Gd^3+^ in GdBaB_9_O_16_. J. Alloys Compd..

[37] Tian Z (2008). Photon cascade emission of Gd^3+^ in Na(Y,Gd)FPO_4_. J. Phys. Chem. C.

[38] Dieke GH, Crosswhite HM (1963). The spectra of the doubly and triply ionized rare earths. Appl. Opt..

[39] Wegh RT, Meijerink A, Lamminmaki RJ, Holsa J (2000). Extending Dieke’’s diagram. J. Lumin..

[40] Wegh RT, Donker H, Meijerink A, Lamminmäki RJ, Hölsä J (1997). Vacuum-ultraviolet spectroscopy and quantum cutting for Gd^3+^ in LiYF_4_. Phys. Rev. B.

[41] Feofilov SP, Seo HJ, Zhou Y, Meltzer RS (2006). Host sensitization of Gd^3+^ ions in yttrium and scandium borates and phosphates: Application to quantum cutting. Phys. Rev. B.

[42] Zhong J (2010). Luminescence properties of NaGd(PO_3_)_4_:Eu^3+^ and energy transfer from Gd^3+^ to Eu^3+^. Appl. Phys. B.

[43] Solé R (2009). Physical properties of self-flux and WO_3_-containing solutions useful for growing type III KGd(PO_3_)_4_ single crystals. J. Cryst. Growth.

[44] Shalapska T (2010). Photon cascade luminescence from Pr^3+^ ions in LiPrP_4_O_12_ polyphosphate. J. Phys. D: Appl. Phys..

[45] Drozdowski W (2008). Scintillation properties of praseodymium activated Lu_3_Al_5_O_12_ single crystals. IEEE Trans. Nucl. Sci..

[46] Trevisani M, Ivanovskikh KV, Piccinelli F, Bettinelli M (2014). Fast 5d-4f luminescence in Pr^3+^-doped K_3_Lu(PO_4_)_2_. J. Lumin..

[47] Jouini A, Gâcon JC, Ferid M, Trabelsi-Ayadi M (2003). Luminescence and scintillation properties of praseodymium poly and diphosphates. Opt. Mater..

[48] Shalapska T (2013). Luminescence properties of Ce^3+^-doped NaPrP_4_O_12_ polyphosphate. J. Phys.: Condens. Matter.

