# Supplementary material for: Single crystal growth, optical absorption and luminescence properties under VUV-UV synchrotron excitation of type III Pr3+:KGd(PO3)4
Source: Sci Rep. 2020 Apr 21;10:6712. doi: 10.1038/s41598-020-63556-w (PMC7174386; doi:10.1038/s41598-020-63556-w)
Supplement: Supplementary file 1 — Supplementary information. [file 41598_2020_63556_MOESM1_ESM.pdf]

Single crystal growth, optical absorption and luminescence properties under VUV-UV synchrotron excitation of type III $Pr^{3+}:KGd(PO_3)_4$

Irina Adell[1], Maria Cinta Pujol[1,*], Rosa Maria Solé[1,*], Matthieu Lancry[2], Nadège Ollier[3], Magdalena Aguiló[1] and Francesc Díaz[1]

[1] Física i Cristal·lografia de Materials i Nanomaterials (FiCMA-FiCNA) - EMaS, Dept. Química Física i Inorgànica, Universitat Rovira i Virgili (URV), Campus Sescelades, c/ Marcel·lí Domingo, 1, E-43007, Tarragona, Spain.

[2] Institut de Chimie Moléculaire et des Matériaux d'Orsay, CNRS-Université Paris Sud, Université de Paris Saclay, Bât.410, 91405 Orsay, France.

[3] Laboratoire des Solides Irradiés, CEA-CNRS-Ecole Polytechnique, Université Paris-Saclay, Palaiseau, France.

* mariacinta.pujol@urv.cat; rosam.sole@urv.cat

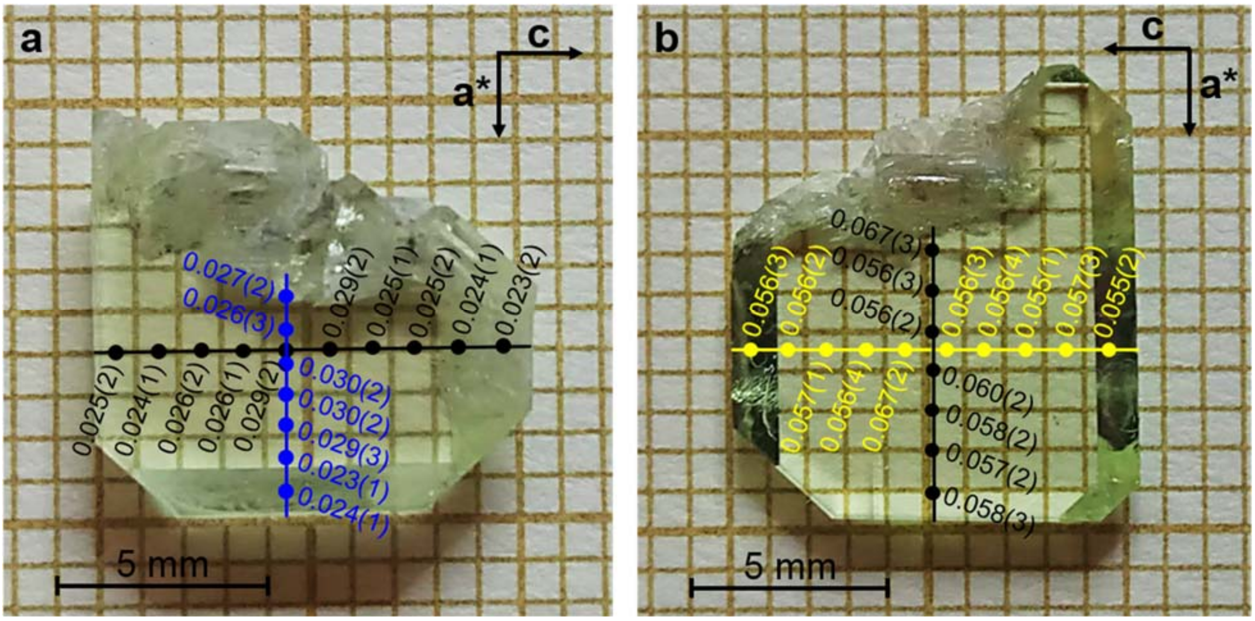

Figure S.1. The [Pr]/([Gd]+[Pr]) atomic ratio in the crystal at several points along the *a** and *c* crystallographic directions in a plate perpendicular to *b* crystallographic axis for (**a**) $KGd_{0.974}Pr_{0.026}(PO_3)_4$ and (**b**) $KGd_{0.942}Pr_{0.058}(PO_3)_4$. The value shown for each dot is the mean Pr^{3+} atomic concentration value of five measurements along with the error of the third decimal in parentheses.

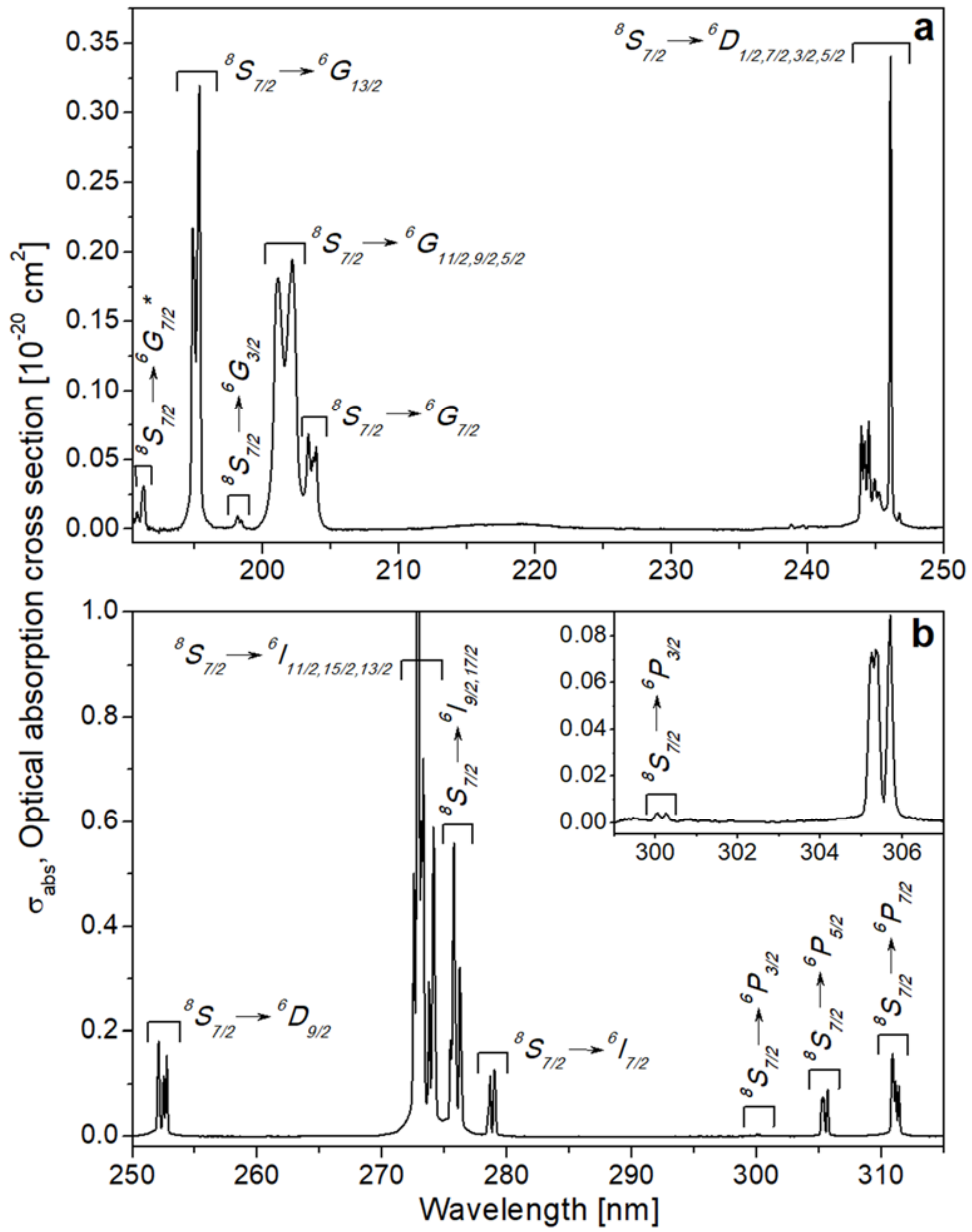

Figure S.2. Unpolarized optical absorption cross section of undoped KGdP at room temperature. Propagation direction is the *b* axis in figures **a** and **b**. All labels indicate the absorption transitions of Gd^{3+}.

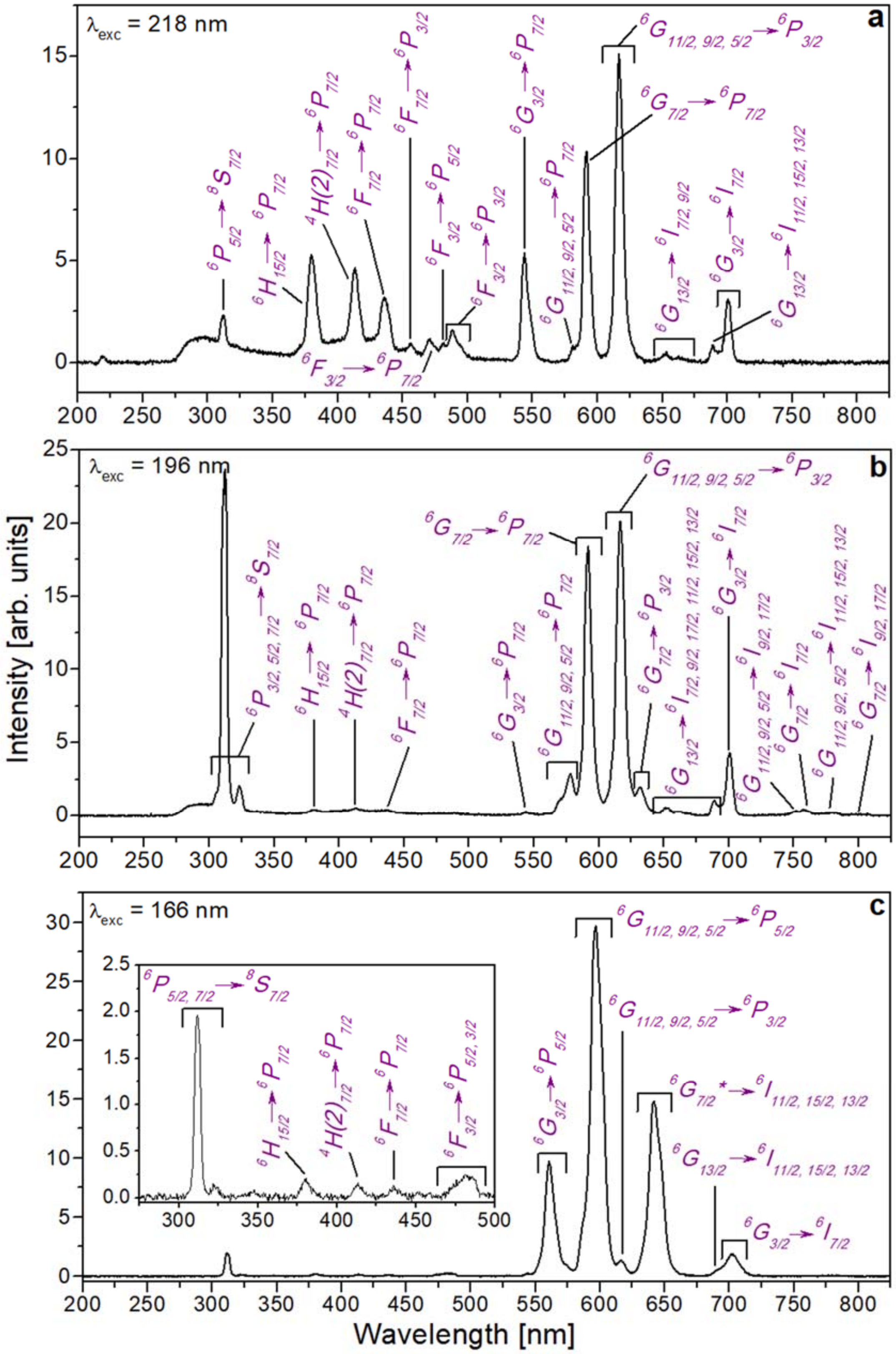

Figure S.3. Optical emission spectra of undoped KGdP under (**a**) 218 nm, (**b**) 196 nm and (**c**) 166 nm excitation. All labels indicate the absorption transitions of Gd^{3+}.
